# Molecular Characteristics of Extended-Spectrum Beta-Lactamases and *qnr* Determinants in *Enterobacter* Species from Japan

**DOI:** 10.1371/journal.pone.0037967

**Published:** 2012-06-15

**Authors:** Hajime Kanamori, Hisakazu Yano, Yoichi Hirakata, Ayako Hirotani, Kazuaki Arai, Shiro Endo, Sadahiro Ichimura, Miho Ogawa, Masahiro Shimojima, Tetsuji Aoyagi, Masumitsu Hatta, Mitsuhiro Yamada, Yoshiaki Gu, Koichi Tokuda, Hiroyuki Kunishima, Miho Kitagawa, Mitsuo Kaku

**Affiliations:** 1 Department of Infection Control and Laboratory Diagnostics, Internal Medicine, Tohoku University Graduate School of Medicine, Sendai, Miyagi, Japan; 2 Department of Clinical Microbiology with Epidemiological Research & Management and Analysis of Infectious Diseases (C-MERMAID), Tohoku University Graduate School of Medicine, Sendai, Miyagi, Japan; 3 Department of Bacteriology, BML, Inc., Kawagoe, Saitama, Japan; 4 Department of Regional Cooperation for Infectious Diseases, Tohoku University Graduate School of Medicine, Sendai, Miyagi, Japan; University of Malaya, Malaysia

## Abstract

The incidence of extended-spectrum β-lactamases (ESBLs) has been increasing worldwide, but screening criteria for detection of ESBLs are not standardized for AmpC-producing Enterobacteriaceae such as *Enterobacter* species. In this study, we investigated the prevalence of ESBLs and/or AmpC β-lactamases in Japanese clinical isolates of *Enterobacter* spp. and the association of plasmid-mediated quinolone resistance (PMQR) determinants with ESBL producers. A total of 364 clinical isolates of *Enterobacter* spp. collected throughout Japan between November 2009 and January 2010 were studied. ESBL-producing strains were assessed by the CLSI confirmatory test and the boronic acid disk test. PCR and sequencing were performed to detect CTX-M, TEM, and SHV type ESBLs and PMQR determinants. For ESBL-producing *Enterobacter* spp., pulsed-field gel electrophoresis (PFGE) was performed using *Xba*I restriction enzyme. Of the 364 isolates, 22 (6.0%) were ESBL producers. Seven isolates of *Enterobacter cloacae* produced CTX-M-3, followed by two isolates producing SHV-12. Two isolates of *Enterobacter aerogenes* produced CTX-M-2. Of the 22 ESBL producers, 21 had the AmpC enzyme, and six met the criteria for ESBL production in the boronic acid test. We found a significant association of *qnrS* with CTX-M-3-producing *E. cloacae*. The 11 ESBL-producing *Enterobacter* spp. possessing *bla*_CTX-M_, *bla*_SHV_, or *bla*_TEM_ were divided into six unique PFGE types. This is the first report about the prevalence of *qnr* determinants among ESBL-producing *Enterobacter* spp. from Japan. Our results suggest that ESBL-producing *Enterobacter* spp. with *qnr* determinants are spreading in Japan.

## Introduction

*Enterobacter* species are an important opportunistic pathogen that can cause nosocomial outbreaks and invasive infections such as bloodstream infections [1]–[3]. The prevalence of Enterobacteriaceae with extended-spectrum β-lactamases (ESBLs) has been increasing worldwide [4]. The prevalence of ESBLs and/or AmpC β-lactamases in clinical isolates of *Enterobacter* spp. from Japan is unknown. The Clinical and Laboratory Standards Institute (CLSI) has established guidelines for detection of ESBLs in *Escherichia coli*, *Klebsiella* spp., and *Proteus mirabilis* [5]. However, there are no recommendations from the CLSI for detection of ESBLs in microorganisms with chromosomal AmpC β-lactamases since the presence of ESBLs can be masked by the AmpC β-lactamases. It has been reported that 3-aminophenylboronic acid (BA) is an inhibitor of AmpC [6], and it is possible to detect ESBLs in AmpC-producing strains by the boronic acid disk test [7].

Quinolone resistance was mainly caused by chromosomal mutations of the quinolone-resistance determining regions in DNA gyrase and DNA topoisomerase IV [8], [9]. Recently, plasmid-mediated quinolone resistance (PMQR) determinants such as *qnr*, *aac(6′)-Ib-cr*, and *qepA*, have been identified in clinical isolates of Enterobacteriaceae worldwide [8], [9]. In addition, the emergence of PMQR in ESBL-producing Enterobacteriaceae is raising public health concerns since the inappropriate use of antimicrobial agents can transfer PMQR genes on the same plasmid as β-lactamase genes [8].

In this study, we investigated the prevalence of ESBLs and/or AmpC β-lactamases in clinical isolates of *Enterobacter* spp. from Japan and the association of PMQR determinants with ESBL-producing isolates. We also evaluated the usefulness of the boronic acid disk test for detecting production of ESBLs by *Enterobacter* spp. This work was presented in part at the 50th Interscience Conference on Antimicrobial Agents and Chemotherapy (ICAAC), Boston, 2010.

**Table 1:** Distribution of antimicrobial resistance genes among ESBL-producing *Enterobacter* spp.

	β-lactamases						PMQR determinants					
ESBL-producing*Enterobacter* spp. (n)	Inducible AmpC	Derepressed AmpC	CTX-M-2	CTX-M-3	TEM-1	SHV-12	*qnrA* *	*qnrB*	*qnrC*	*qnrS*	*qepA*	*aac(6')-Ib-cr*
*E. cloacae* (18)	1	16	0	7	9	2	2	0	0	7	0	0
*E. aerogenes* (4)	1	3	2	0	1	0	0	0	0	1	0	0

* All *qnrA* were *qnrA1*.

**Table 2:** Profile of *Enterobacter* spp. carrying ESBL genes and results of the boronic acid disk test.

StrainNo.	Sample	Organism	AmpC	ESBL	PMQR	PFGEtype	MIC (mg/L)							ESBL confirmatory test	
							CTX	CAZ	FOX	FEP	MEM	LVX	AMK	without BA	with BA
A33	pus	*E. aerogenes*	derepressed	CTX-M-2	notdetected	EA1	≥128	≥128	≥64	32	2	1	≤8	positive	positive
D35	urine	*E. cloacae*	derepressed	SHV-12	*qnrA1*	EC3	≥128	≥128	≥64	≥64	8	16	16	positive	positive
F8	pus	*E. aerogenes*	inducible	CTX-M-2	*qnrS*	EA2	≥128	8	≥64	≥64	1	1	≤8	positive	positive
F10	sputum	*E. cloacae*	inducible	CTX-M-3	*qnrS*	EC4	≥128	8	≥64	≥64	≤0.5	8	≤8	positive	positive
F22	sputum	*E. cloacae*	derepressed	CTX-M-3	*qnrS*	EC1	≥128	≥128	≥64	≥64	2	16	≤8	negative	positive
F25	sputum	*E. cloacae*	derepressed	CTX-M-3	*qnrS*	EC1	≥128	≥128	≥64	≥64	2	16	≤8	negative	positive
F33	sputum	*E. cloacae*	derepressed	CTX-M-3	*qnrS*	EC1	≥128	≥128	≥64	≥64	1	16	≤8	negative	positive
F38	sputum	*E. cloacae*	derepressed	CTX-M-3	*qnrS*	EC1	≥128	≥128	≥64	≥64	2	16	≤8	negative	positive
F43	sputum	*E. cloacae*	derepressed	CTX-M-3	*qnrS*	EC2	≥128	64	≥64	≥64	≤0.5	16	≤8	negative	positive
F46	pus	*E. cloacae*	derepressed	CTX-M-3	*qnrS*	EC1	≥128	≥128	≥64	≥64	1	8	≤8	Negative	positive
I62	urine	*E. cloacae*	derepressed	SHV-12	*qnrA1*	EC3	≥128	≥128	≥64	32	2	2	≤8	Positive	positive

CTX: cefotaxime, CAZ: ceftazidime, FOX: cefoxitin, FEP: cefepime, MEM: meropenem, LVX: levofloxacin, AMK: amikacin, BA: 3-aminophenylboronic acid. EC: *Enterobacter cloacae*, EA: *Enterobacter aerogenes.*

**Figure 1:**
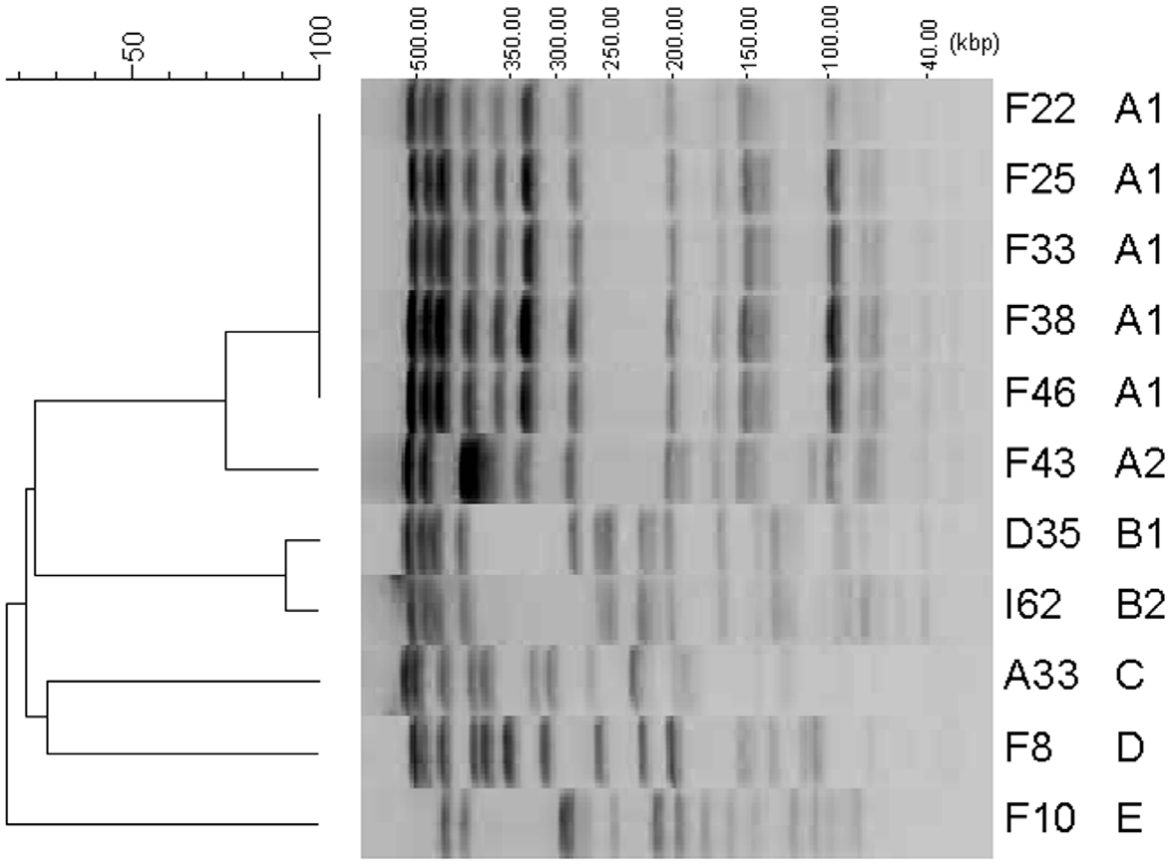
Dendrogram and PFGE of *Xba*I-digested genomic DNAs from ESBL-producing *Enterobacter* species. EC: *Enterobacter cloacae*, EA: *Enterobacter aerogenes*. Strain No. and PFGE type correspond to those in Table 2.

## Materials and Methods

### Bacterial Strains

We studied a total of 364 consecutive and nonduplicate clinical isolates of *Enterobacter* spp., consisting of 228 isolates of *Enterobacter cloacae* and 136 isolates of *Enterobacter aerogenes*. These isolates were collected between November 2009 and January 2010 from 10 regions of Japan, including 38 isolates from Hokkaido, 13 from Miyagi, 19 from Niigata, 95 from Tokyo, 7 from Aichi, 50 from Osaka, 15 from Hiroshima, 10 from Ehime, 99 from Fukuoka, and 18 from Okinawa. *Enterobacter* spp. were identified by the Vitek-2 System (Sysmex-bioMérieux Japan, Tokyo). The isolates included 115 from urine (31.6%), 95 from sputum (26.1%), 77 from throat swabs (21.2%), 26 from nasal (7.1%), and 51 from other specimens (14.0%). Ethical approval was not needed according to the ethical guidelines for epidemiological research by the Japanese government because this study focused on bacterial aspects.

### Antimicrobial Susceptibility Testing

Antimicrobial susceptibility testing of ESBL-producing isolates was performed with a frozen plate (Eiken Chemical Co., Ltd., Tokyo, Japan) and the broth microdilution method described by the CLSI [10]. The following antimicrobial agents were tested: ampicillin (range: 1–32 mg/L), piperacillin (4–128 mg/L), piperacillin-tazobactam (4/4–128/4 mg/L), cefoxitin (1–32 mg/L), cefpodoxime (2–64 mg/L), cefotaxime (2–64 mg/L), ceftazidime (2–64 mg/L), cefepime (1–32 mg/L), meropenem (0.5–16 mg/L), aztreonam (1–32 mg/L), ciprofloxacin (0.5–16 mg/L), levofloxacin (0.5–16 mg/L), gentamicin (1–16 mg/L), amikacin (8–64 mg/L), and trimethoprim-sulfamethoxazole (2/38–4/76 mg/L). The CLSI interpretive criteria were employed for each antibacterial agent [5]. Quality control was conducted by using *E. coli* ATCC 25922 and *E. coli* ATCC 35218.

### Phenotypic Detection of AmpC β-lactamases and Extended-spectrum β-lactamases

According to the characteristics of β-lactamase production, AmpC-producing stains with a zone diameter ≤14 mm for cefoxitin were classified as derepressed AmpC mutants when the zone diameter was ≤17 mm for cefpodoxime/clavulanic acid, while inducible AmpC-producing strains had a zone ≥18 mm for cefpodoxime/clavulanic acid and a positive cefoxitin/cefpodoxime antagonism test [7], [11]. Possible ESBL-producing strains were screened by a zone diameter ≤20 mm for cefpodoxime, followed by the CLSI confirmatory test for ESBLs using disks of ceftazidime (30 µg), cefotaxime (30 µg), and cefpodoxime (10 µg) with or without clavulanic acid (10 µg) [5]. The boronic acid disk test was also performed to detect ESBL production using disks with 400 µg of boronic acid, as described previously [11]. A ≥5 mm increase of the zone diameter was considered to be a positive result that indicated the presence of ESBLs.

### ESBL and PMQR Gene Detection and Sequencing

PCR was performed to identify various resistance genes, including β-lactamase genes (*bla*_TEM_, *bla*_SHV_ and *bla*_CTX-M_), PMQR genes (*qnrA*, *qnrB*, *qnrC*, *qnrS*, and *qepA*), and *aac(6')-Ib* [12], [13], [14]. For CTX-M positive strains, the CTX-M group was determined by PCR using CTX-M-1, CTX-M-2, and CTX-M-9 group-specific primers [15], [16], [17]. For positive controls, we used DNA extracts from clinical isolates which were confirmed to possess β-lactamase or PMQR genes by sequencing. PCR products were purified by using a QIA quick PCR Purification Kit (Qiagen K. K., Tokyo, Japan), and then were sequenced by using specific primers [14], [15], [16], [17] with an ABI BigDye Terminator v3.1 Cycle Sequencing Kit (Applied Biosystems, Foster City, CA) on an ABI 3730×l Analyzer (Applied Biosystems). Sequencing data were analyzed with BLAST version 2.2.24 (http://blast.ddbj.nig.ac.jp/top-j.html).

### Pulsed-field Gel Electrophoresis

For 11 ESBL-producing isolates (nine isolates of *E. cloacae* and two isolates of *E. aerogenes*) in which *bla*_CTX-M_, *bla*_SHV_, or *bla*_TEM_ were identified by PCR, evaluation of chromosomal polymorphisms was done by pulsed-field gel electrophoresis (PFGE) using the *Xba*I restriction enzyme (Takara Bio Inc., Otsu, Japan), as described previously [18]. Electrophoresis was performed on 1% PFGE agarose gel with a CHEF-DR III system (Bio-Rad Laboratories, Richmond, CA, USA). Electrophoretic patterns were analyzed with GelCompar II version 3.0 (Applied Maths, Kortrijik, Belgium). Similarity between two tracks was calculated by using the coefficient of Jaccard and the band positions. Cluster analysis was performed by the unweighted pair-group method using arithmetic averages, and a dendrogram was generated by the software. Isolates with ≥80% similarity were considered to reside within a single cluster.

### Statistical Analysis

Statistical analysis was conducted with the chi-square test or Fisher’s test. A *P* value of less than 0.05 was considered significant.

## Results and Discussion

A total of 206 (90.4%) of the 228 isolates of *E. cloacae* and 130 (95.6%) of the 136 isolates of *E. aerogenes* were phenotypically confirmed to be AmpC producers. Among the 228 *E. cloacae* isolates, there were 60 derepressed AmpC mutants (26.3%) and 146 inducible AmpC producers (64%), while the 136 *E. aerogenes* isolates included 21 derepressed AmpC mutants (15.4%) and 109 inducible AmpC producers (80.1%). We found that 18 (7.9%) of the 228 isolates of *E. cloacae* and 4 (2.9%) of the 136 isolates of *E. aerogenes* were positive in the ESBL confirmatory test. All of the ESBL-producing isolates, except one *E. cloacae* isolate, also produced AmpC β-lactamases. In Osaka prefecture, the prevalence of ESBL-producing *E. cloacae* (8/35) was significantly higher than that for all of Japan (18/228) (*P*<0.05). The prevalence of ESBL-producing *Enterobacter* spp. varies among countries and regions, as well as between detection methods. It was previously reported that ESBL-producing *E. cloacae* had a prevalence of 17.7% in Algeria [19], 28% in Taiwan [20], and 35.4% in Korea [21]. Our data suggest that although the prevalence of ESBL-producing *E. cloacae* in Japan is still lower than in those countries, nevertheless the spread of ESBL-producers is certainly occurring at large in our country.

Of the 22 ESBL producers, 16 isolates (72.7%) were only detected by the CLSI confirmatory test, while six isolates (27.3%) met the criteria for ESBL production in the boronic acid test. Jeong et al. reported that the CLSI confirmatory test only detected 72.1% of ESBL producers, while the boronic acid disk test detected 98.4% and showed no false-positive results [7]. In the present study, the boronic acid disk test was useful for detecting ESBL producers among chromosomal AmpC-producing *Enterobacter* spp.

All of the ESBL-producing *Enterobacter* spp. were resistant to ampicillin and piperacillin. Nineteen isolates (86.4%) and 22 isolates (100%) were resistant to piperacillin-tazobactam and cefoxitin, respectively, suggesting a high prevalence of AmpC β-lactamases among ESBL producers. Resistance to cefpodoxime, cefotaxime, ceftazidime, cefepime, and meropenem was shown by 22 (100%), 22 (100%), 20 (90.9%), 13 (59.1%), and 0 of these isolates, respectively. Thus, carbapenem may be the most effective treatment for ESBL-producing *Enterobacter* spp. Thirteen isolates (59.1%) and 21 isolates (95.5%) were susceptible to gentamicin and amikacin, respectively. Of the 10 *qnr*-positive isolates, 8 (80%) were resistant to both ciprofloxacin and levofloxacin, while only one (8.3%) of the 12 *qnr*-negative isolates was resistant to both ciprofloxacin and levofloxacin (*P*<0.01). *qnr* determinants can confer weak quinolone resistance [22]. There is concern that Enterobacteriaceae possessing *qnr* and lacking sufficient chromosomal quinolone resistance could be classified as susceptible to fluoroquinolones according to the CLSI criteria.

The distribution of antimicrobial resistance genes among ESBL-producing *Enterobacter* spp. is summarized in Table 1. Of the 22 ESBL-producing strains, nine were positive for CTX-M, two for SHV, and 10 for TEM by PCR. Sequencing revealed that seven of the 20 isolates of *E. cloacae* had CTX-M-3, suggesting that it is predominant among *E. cloacae* in Japan. An outbreak of CTX-M-3-producing *E. cloacae* infection arising from a patient with immature teratoma in the pediatric ward of a university hospital in Osaka prefecture has been described previously [2]. Two of the four isolates of *E. aerogenes* produced CTX-M-2. To our knowledge, this is the first report of CTX-M-2 among *E. aerogenes* in Japan. Both of the two SHV-positive isolates and all 10 TEM-positive isolates had SHV-12 and TEM-1, respectively.

Of the 22 ESBL-producing strains, 10 were positive for PMQR determinants (45.5%). Of the 10 isolates possessing PMQR genes, eight and two were positive for *qnrS* and *qnrA*, respectively. This is also the first report about *qnr* determinants among ESBL-producing *Enterobacter* spp. from Japan. The profile of *Enterobacter* spp. carrying ESBL genes is shown in Table 2. All seven of the CTX-M-3-producing *E. cloacae* strains had *qnrS*, and *qnrS* positivity was significantly more common among CTX-M-3-producing *E. cloacae* (7/7) compared with other ESBL producers (7/7 vs. 1/4, *P*<0.05). Both of the two SHV-12-producing *E. cloacae* possessed *qnrA1*. None of the 22 ESBL-producing *Enterobacter* spp. had *qnrB*, *qnrC*, *qepA*, and *aac(6')-Ib-cr*. An association of PMQR determinants with ESBLs has been reported by several authors. Park et al. reported a high prevalence of *qnrA* and *qnrB* among ESBL-producing *E. cloacae* in Korea [23], while there was a close association of *qnrA*, *qnrB*, or *qnrS* with SHV-12 production by *E. cloacae* in France, Algeria, and Taiwan [19], [20], [24]. An association of *qnrB* or *qnrS* with CTX-M-15 production by *E. cloacae* was also reported in Algeria [19].

Of the 22 ESBL-producing isolates which were phenotipically identified by disk tests, 11 isolates (nine for *E. cloacae* and two for *E. aerogenes*) possessed ESBL genes (*bla*_CTX-M-2_, *bla*_CTX-M-3_, or *bla*_SHV-12_), but the remaining 11 isolates did not. Dissociation between disk tests and PCR detection results can occur since screening criteria for detection of ESBLs are not standardized for *Enterobacter* spp. Therefore, we focused on 11 isolates in which ESBL genes were identified by PCR and performed PFGE for them. Figure 1 shows dendrogram and PFGE of *Xba*I-digested genomic DNAs from 11 ESBL-producing *Enterobacter* spp. The 11 ESBL-producing isolates possessing *bla*_CTX-M_, *bla*_SHV_, or *bla*_TEM_ were divided into six unique PFGE types. Of seven isolates possessing *bla*_CTX-M-3_ and *qnrS* from Osaka, five had identical PFGE type (EC1), while two had different types (EC2 and EC4). Two isolates possessing both *bla*_SHV-12_ and *qnrA1* from Tokyo and Fukuoka were identical cluster (EC3). These results suggest that *E. cloacae* harbouring both *bla*_CTX-M-3_ and *qnrS* or both *bla*_SHV-12_ and *qnrA1* are spreading via plasmids or transposons in Japan, although a limitation of our study is the relatively small sample size. Careful and continuous monitoring of antimicrobial resistance among *Enterobacter* spp. is needed.
